# Early postnatal exposure to isoflurane causes cognitive deficits and disrupts development of newborn hippocampal neurons via activation of the mTOR pathway

**DOI:** 10.1371/journal.pbio.2001246

**Published:** 2017-07-06

**Authors:** Eunchai Kang, Danye Jiang, Yun Kyoung Ryu, Sanghee Lim, Minhye Kwak, Christy D. Gray, Michael Xu, Jun H. Choi, Sue Junn, Jieun Kim, Jing Xu, Michele Schaefer, Roger A. Johns, Hongjun Song, Guo-Li Ming, C. David Mintz

**Affiliations:** 1 Institute for Cell Engineering, The Johns Hopkins University School of Medicine, Baltimore, Maryland, United States of America; 2 Department of Neurology, The Johns Hopkins University School of Medicine, Baltimore, Maryland, United States of America; 3 Department of Anesthesiology, The Johns Hopkins University School of Medicine, Baltimore, Maryland, United States of America; 4 The Solomon Snyder Department of Neuroscience, The Johns Hopkins University School of Medicine, Baltimore, Maryland, United States of America

## Abstract

Clinical and preclinical studies indicate that early postnatal exposure to anesthetics can lead to lasting deficits in learning and other cognitive processes. The mechanism underlying this phenomenon has not been clarified and there is no treatment currently available. Recent evidence suggests that anesthetics might cause persistent deficits in cognitive function by disrupting key events in brain development. The hippocampus, a brain region that is critical for learning and memory, contains a large number of neurons that develop in the early postnatal period, which are thus vulnerable to perturbation by anesthetic exposure. Using an in vivo mouse model we demonstrate abnormal development of dendrite arbors and dendritic spines in newly generated dentate gyrus granule cell neurons of the hippocampus after a clinically relevant isoflurane anesthesia exposure conducted at an early postnatal age. Furthermore, we find that isoflurane causes a sustained increase in activity in the mechanistic target of rapamycin pathway, and that inhibition of this pathway with rapamycin not only reverses the observed changes in neuronal development, but also substantially improves performance on behavioral tasks of spatial learning and memory that are impaired by isoflurane exposure. We conclude that isoflurane disrupts the development of hippocampal neurons generated in the early postnatal period by activating a well-defined neurodevelopmental disease pathway and that this phenotype can be reversed by pharmacologic inhibition.

## Author summary

The United States Food and Drug Administration has recently warned that exposure to anesthetic and sedative drugs during the third trimester of prenatal development and during the first 3 years of life may cause lasting impairments in cognitive function. The mechanisms by which this undesirable side effect occurs are unknown. In this manuscript, we present evidence in mice that early developmental exposure to isoflurane, a canonical general anesthetic, disrupts the appropriate development of neurons in the hippocampus, a brain region associated with learning and memory. Isoflurane also causes up-regulation of the mechanistic target of rapamycin (mTOR) pathway, a signaling system that has been associated with other neurodevelopmental cognitive disorders. Treatment with an inhibitor of the mTOR pathway after isoflurane exposure normalizes neuronal development and also ameliorates the impairments in learning induced by isoflurane. We conclude that early exposure to isoflurane can cause learning deficits via actions on the mTOR pathway, and that this mechanism represents a potentially druggable target to minimize the side effects of anesthetics on the developing brain.

## Introduction

Several large retrospective analyses link exposure to anesthetics and surgery within the first 3 years of life with subsequent effects on cognitive function, as measured by worsened performance on school assessments, an increase in billing codes relevant to learning disorders, and deficits in neuropsychological testing [1–3]. It is difficult to separate the effects of surgery, anesthesia, and comorbidity in clinical studies. However, multiple independent investigations conducted in rodent models using different anesthetics and varying exposure paradigms in the absence of surgery indicate that early developmental exposure to general anesthetic agents results in lasting impairment on behavioral measures of neurocognitive function, predominantly in the domain of learning and memory [4–12]. While 2 recent clinical studies give some reassurance that short, single exposures in healthy children may not have dramatic consequences [13,14], clear evidence of lasting cognitive deficits was detected recently in a carefully conducted study of a somewhat longer clinically relevant anesthetic exposure in nonhuman primates [15]. Thus, there are serious concerns in the anesthesiology, surgery, and pediatrics literature that anesthetic exposure may result in worsened cognitive outcomes for some unknown fraction of the hundreds of thousands of children under age 4 who undergo surgery each year [16–18]. In response to these findings, the US Food and Drug Administration recently issued a drug safety communication warning that anesthetic exposure may pose risks to brain development and calling for further research on this topic. The molecular and cellular mechanisms underlying this phenomenon have yet to be clearly elucidated, and no prophylactic or treatment strategies exist.

Much of the literature on the effects of anesthetic exposure on brain function focuses on the potential for anesthetics to activate apoptotic cell death pathways in neurons [6,19], but more recent work has led to the novel hypothesis that anesthetics cause lasting effects on cognitive function via sublethal effects on critical processes in neuronal development [20]. In humans, the neural circuitry underlying higher brain functions, such as learning, is primarily established between the second trimester and early childhood [21], a period that includes the window of putative vulnerability to anesthetics identified in epidemiologic studies [18]. During this time, critical ongoing developmental events are occurring in many neurons of the hippocampus, including growth of dendritic arbors and generation of dendritic spines, which are the postsynaptic elements of excitatory synapses [22]. There are substantial differences in developmental timelines in the different species in which the effects of early postnatal anesthesia exposure on cognitive function have been studied, but one notable common feature is the generation and development of a large percentage of the dentate gyrus granule cell (DGC) neurons in the hippocampus [23], a structure that is critical to cognitive functions, including learning and memory. Thus, in this study we investigated the effects of anesthesia exposure on dendritic arbor and spine development in early postnatally generated DGCs, which may be an important target population and may also serve as a model for postnatal neuron development in other brain regions.

We employed a retrovirus-mediated labeling method in intact mice to examine the development of dendrite arbors and dendritic spines in DGCs in vivo after exposure to a clinically relevant dose of isoflurane. This approach allows morphological analyses of a uniform and well-studied population of neurons, the DGCs, at a single cell level in vivo [24]. We find that early postnatal exposure to isoflurane results in a substantial and lasting disruption of dendritic arborization and spine development. Isoflurane was found to over-activate the mechanistic target of rapamycin (mTOR) pathway, a signaling system critical for normal development, which has been implicated in neurodevelopmental disorders in which cognitive function is affected, including autism and fragile X mental retardation [25,26]. Strikingly, the adverse effects of isoflurane on both dendrite morphology and behavioral tests of learning can be reversed with rapamycin, an mTOR inhibitor. Our findings reveal a novel mechanism by which anesthetics disrupt brain development that has been implicated in other neurodevelopmental disorders and that is potentially reversible via drug therapy.

## Results and discussion

**Fig 1:**
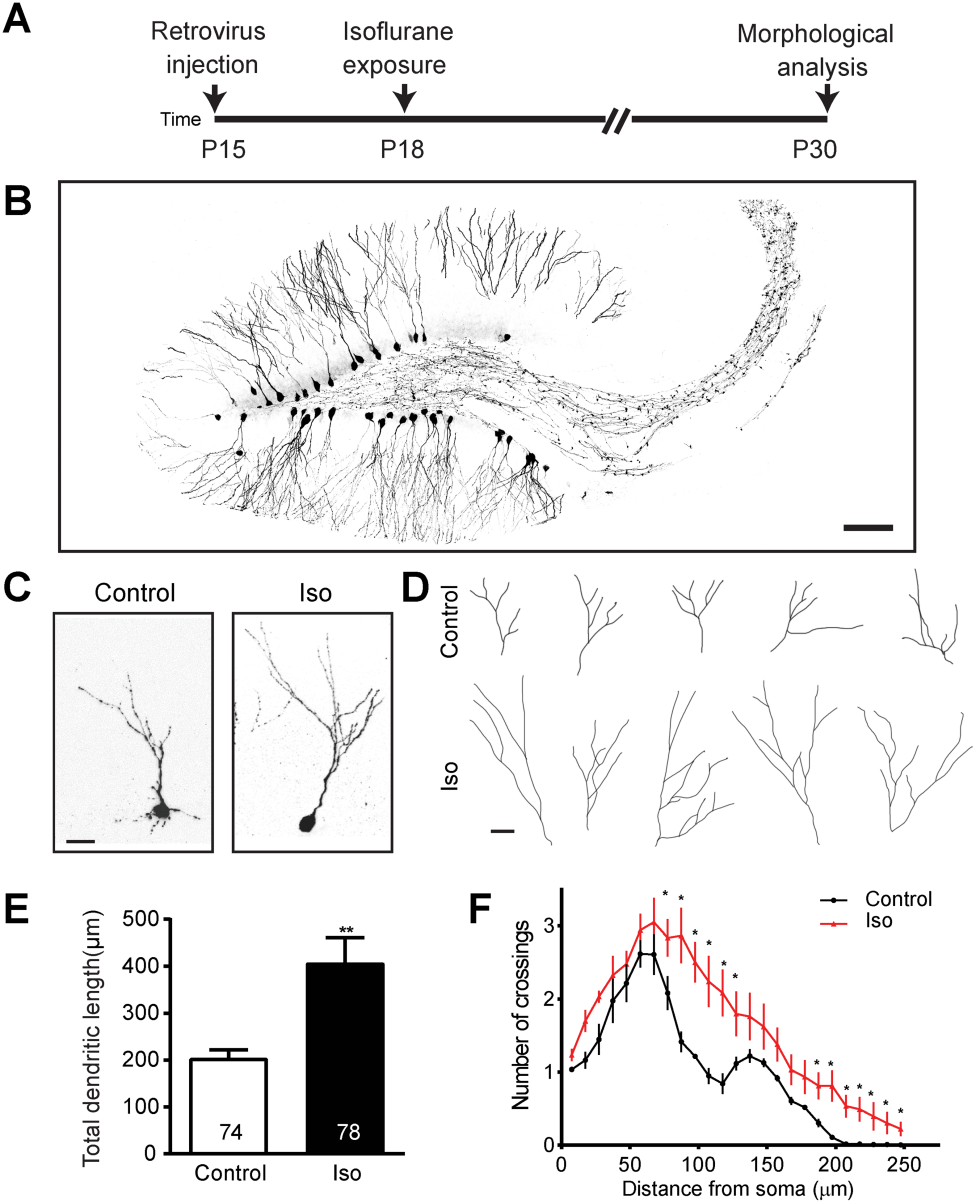
Isoflurane exposure results in overgrowth of dendritic arbors. (A) A schematic diagram of isoflurane exposure procedure for morphology examination. (B) Sample confocal image of dentate gyrus granule cell (DGCs) infected with retrovirus expressing green florescent protein (GFP) (scale bar: 100 μm). Representative confocal images (C) and tracings (D) of individual control and isoflurane-exposed GFP+ neurons at postnatal day (P) 30 exhibiting overgrowth in the isoflurane group relative to control conditions (scale bar: 10 μm for both C and D). Summaries of total dendritic length (E) and Sholl analysis of dendritic complexity (F) of GFP+ neurons show marked overgrowth of dendritic arbors. Numbers associated with bar graph indicate the number of neurons examined from at least 5 animals per group. The same groups of neurons were examined in (E) and (F). Values represent mean ± SEM (**p < 0.01; Student *t* test for E and *p < 0.0001 ANOVA for F). Underlying data in S1 Data under Fig 1F tab.

In order to investigate the effects of anesthetics on dentate gyrus neuron development in vivo, we employed stereotaxic injection to deliver a retrovirus expressing green florescent protein (GFP) to label newly generated dentate gyrus neurons [24]. Injections were conducted at postnatal day (P) 15; on P18, the animals were exposed to isoflurane, a canonical halogenated ether vapor anesthetic. The dose of isoflurane exposure (1.5%) falls well within clinically relevant parameters, as the minimum alveolar concentrations of isoflurane ranges between 1.6% and 1.8% in children between ages 0 and 4 [27]. A 4 hour-exposure duration was selected based on clinical data, which showed that significant learning deficits in children are associated with more than 2 hours of anesthetic exposure [3]. All exposed mice survived and recovered readily, and results of physiologic monitoring of sentinel animals are shown in S1 Table. Tissue was collected for morphological studies at P30. A flow diagram of these experiments is shown in Fig 1A.

We sought to determine whether exposure to anesthetics during development alters neuronal structure in newborn DGCs lasting fashion. Previous investigations have been potentially confounded by an inability to determine the developmental stage at which any given neuron under analysis was affected by anesthetics, given the nonhomogenous timeline of neuronal development that occurs even within discrete brain regions. In our model, the labeled DGCs, which have fully definable structure due to GFP expression that allows for easy analysis of morphology (Fig 1B), represent a cohort of cells with a uniform birthdate, all of which were exposed to anesthetics at the same point in their developmental timeline. Examination of dendritic structure revealed a striking finding: compared to neurons in unexposed littermate controls, labeled neurons in isoflurane-exposed animals exhibit an 83% increase in total dendritic arbor length at P30 (*p* < 0.005; Fig 1C–1E). To further elucidate this phenomenon, we conducted a Sholl analysis, which revealed a significant increase in dendrite arbor complexity with isoflurane exposure (*p* < 0.0001; Fig 1F). This finding seems to represent an acceleration of dendrite growth, because dendritic length and complexity in the isoflurane group no longer differs from controls at P60 (S1A–S1D Fig). Branch number is unaffected at either time point (S1E Fig). Cell positioning within the dentate gyrus is unaffected (S1F Fig), suggesting no deficits in migration, but soma size is significantly increased with isoflurane exposure at P30, but not P60 (S1G Fig), further suggesting an abnormal acceleration in DGC growth.

The change in timing of dendritic development resulting from anesthetic exposure represents a novel and surprising effect of anesthetics on the developing brain. In vitro studies of axon growth suggest that volatile anesthetics such as isoflurane may slow the growth of axons and prepolarized neurites [28,29], but axons and dendrites have substantial differences in their developmental properties [30]. A cell culture study that specifically examined dendrites found that exposure to propofol, but not midazolam, at 1 day in vitro (DIV) caused a lasting suppression of dendritic growth in GABAergic neurons [31]. While the timing of exposure and measurement loosely resembles our model, the difference in anesthetic agents and the lack of an in vivo context may explain the disparate findings. Furthermore, the DGCs are primarily glutamatergic and have properties quite distinct from the GABAergic interneurons population [32]. The only other study to assess the effects of anesthetics on dendrites in vivo found no acute change in the dendritic arbors of prefrontal cortex pyramidal neurons in P16 rats 6 hours after isoflurane exposure, but did not examine longer-term effects [33]. Thus, it is unclear whether the transient dendritic hypertrophy we observed might generalize beyond the DGCs exposed early in their development. Abnormalities in dendritic arbor development may have a profound impact on the function of a neuron via effects on the neuron’s synaptic field and pathologic overgrowth of dendrites has been hypothesized as a component of human neurodevelopmental diseases such as autism and schizophrenia [34]. Overgrowth of dendritic arbors has been observed in some animal models of Fragile-X syndrome [35] and autism [36]. However, we cannot determine whether the phenomenon that we observed is a cause of neuronal dysfunction or simply an epiphenomenon or adaptive response.

**Fig 2:**
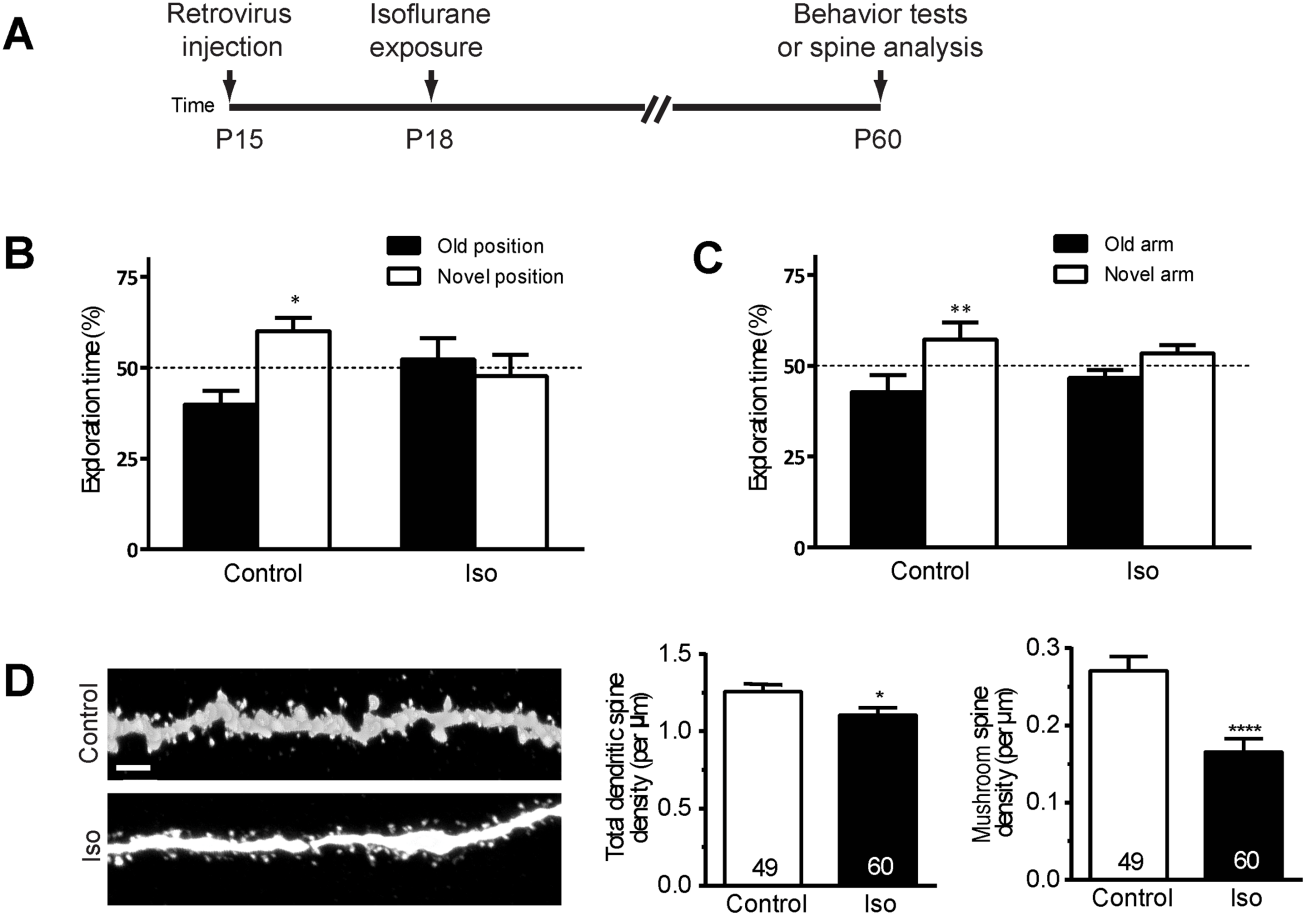
Isoflurane exposure impairs spatial learning and causes a loss of dendritic spines in dentate gyrus neurons. (A) A schematic diagram of isoflurane exposure procedure for behavior tests and spine analysis. Shown in (B and C) are summaries of the object-place recognition test (B) and the Y-maze test (C) (Control *n* = 12, Isoflurane *n* = 11; **p < 0.01, Student *t* test). (D) Representative processed confocal images of dendritic spines of control and isoflurane-exposed green florescent protein positive (GFP) neurons at postnatal day (P) 60 (scale bar: 2 μm). Shown on right are summary plots of total and mushroom class dendritic spine density, revealing a striking loss of mature spines. Numbers associated with the bar graph indicate the number of dendritic segments examined from at least 5 mice from each group, a total of 2,586 spines in the control group and 2,818 spines in the isoflurane group were analyzed (*p < 0.05; ****p < 0.0001, Student *t* test). Underlying data in S1 Data under Fig 2B-D tab.

We next asked whether isoflurane exposure results in long-term deficits in learning potentially attributable to a disruption of the function of the DGCs in which we have detected a morphological abnormality. Animals were exposed to isoflurane 1.5% for 4 hours at P18 and evaluated for deficits in the object-place recognition and the Y-maze tests of spatial learning at P60 (Fig 2A). Both of these tasks are highly sensitive to alterations in the function of even small numbers of dentate gyrus neurons [37]. In the object-place recognition test, control animals spend significantly more time exploring objects in novel positions, but isoflurane-exposed animals exhibit no exploration preference (Fig 2B, S2A and S2C Fig). Similarly, in the Y-maze test, unlike controls, isoflurane-exposed mice do not exhibit a preference for exploration of the newly available arm (Fig 2C, S2B and S2D Fig). These data demonstrate that isoflurane exposure results in a lasting reduction in performance on the tasks of spatial learning that are dependent on the hippocampus and potentially sensitive to disruption of the development of the dentate gyrus.

Next, we asked whether the observed changes in behavior after anesthetic exposure could be attributed to a lasting change in synapses of the DGCs. We used the retrovirus-mediated labeling method to quantify the density of dendritic spine formations at P60, the age at which behavioral testing took place (Fig 2A). Dendritic spines are dynamic, actin-dependent structures that are critical for learning and memory functions [38]. Spines have range of morphologies traditionally classified as stubby, thin, and mushroom shape. We found a small, but significant decrease in the total density of spines (12% decrease, *p* < 0.05) in anesthesia-exposed groups, and a very striking 39% decrease in the density of mushroom spines (*p* < 0.001; Fig 2D, S2E and S2F Fig). No significant change was seen in the density of stubby or thin spines (S2G and S2H Fig). Stubby spines are thought to be immature, thin spines are highly plastic and often transient unless converted into mushroom morphology, and mushroom spines typically represent long-lasting, stable synaptic connections [39]. The reduction in mushroom spine number suggests a substantial loss of synapses that could reasonably account for the reduced performance in spatial learning.

Our finding of a reduction in spine density in the cohort of labeled DGCs is in keeping with an increasing body of work suggests that relatively immature neurons exposed to anesthetics may suffer a long-lasting loss of synaptic connections. Studies from 2 different groups in rats found that early postnatal exposure to either sevoflurane alone or a combination of isoflurane, midazolam, and nitrous oxide resulted in a long-term reduction in the number of synaptic profiles measured by quantitative electron microscopy in the hippocampal CA1 and subiculum areas, respectively [40,41]. The hippocampus is a relatively late developing structure [42], and thus during early postnatal life, it has numerous neurons that are still undergoing active dendrite arborization and spine formation. In support of the hypothesis that developing neurons may be vulnerable to anesthesia-induced synapse loss, a long-term study of the effects of single dose propofol exposure in rats found a decrease in spines in the medial prefrontal cortex of rats exposed at P5 and measured at P90 [43]. In striking contrast, exposure at P15 actually caused an increase in spine number [43], suggesting a notable difference in vulnerability that occurs with neuronal maturation. If developmental exposure to anesthetics can cause a lasting or even permanent loss of synaptic connections in key brain regions such as the hippocampus and pre-frontal cortex this event may represent a perturbation of the development of key brain circuitry, which, in turn, could explain an ongoing loss of cognitive function.

**Fig 3:**
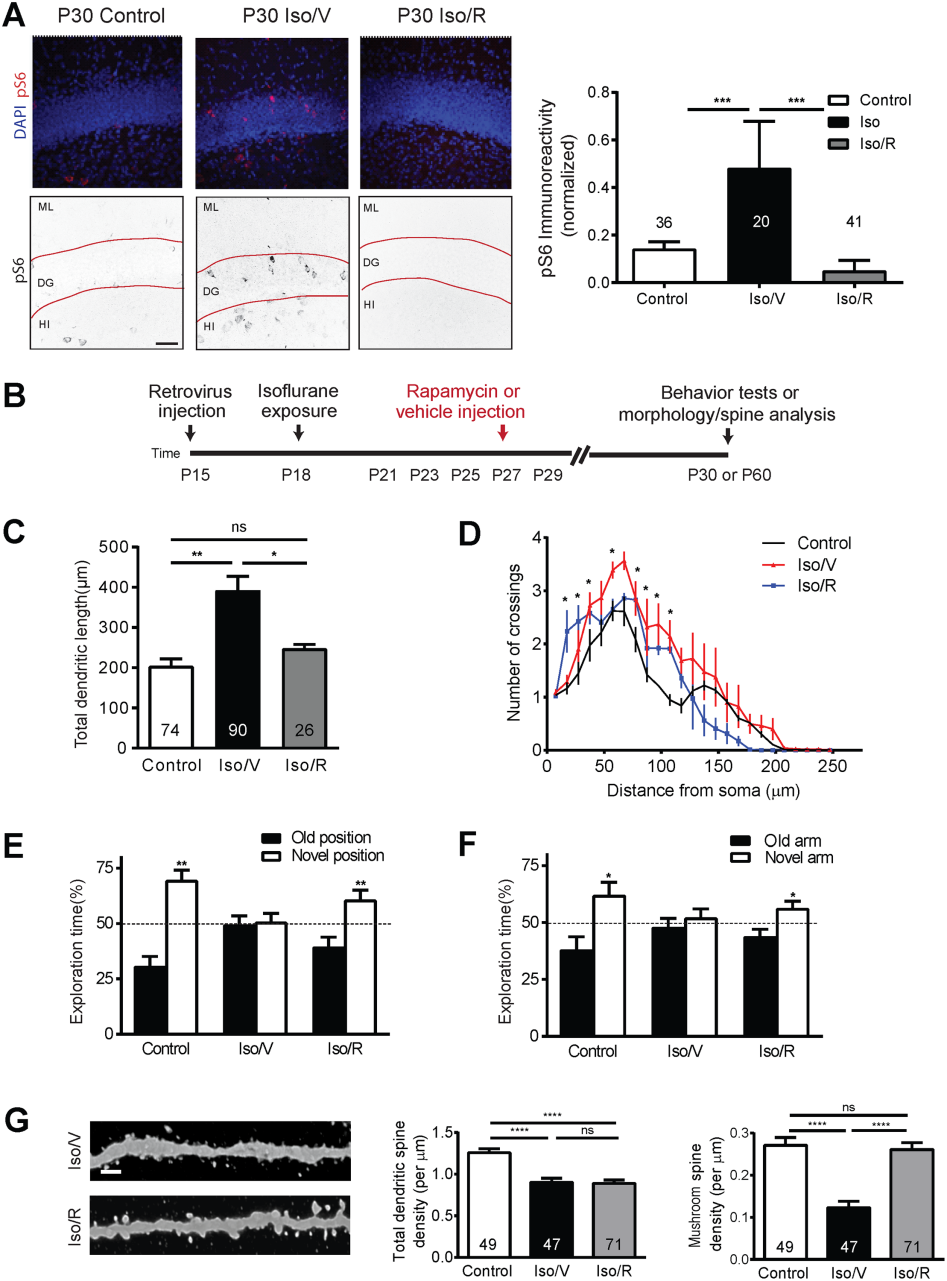
Isoflurane exposure leads to aberrant activation of the mechanistic target of rapamycin (mTOR) signaling pathway, and pharmacological inhibition of the mTOR activities rescues deficits in behavioral tests and loss of spines. (A) Representative confocal images of phospho-S6 (pS6) immunofluorescence at postnatal day (P) 30 in the dentate gyrus showing an increase in labeling in the isoflurane plus vehicle (Iso/V) group relative to controls and a return to baseline in the group exposed to isoflurane and subsequently treated with rapamycin, designated Iso/R. The upper panels are original confocal images with DAPI in blue and pS6 labeling in red, and the lower panels are processed for quantification with black pS6 signal on white background (ML, molecular layer; DG, dentate gyrus; HI, hilus, scale bar: 50 μm). Also shown in (A) quantification of normalized pS6 expression in the dentate gyrus granule cell layer (***p < 0.001, ANOVA, numbers in each bar represent n for images analyzed). (B) Schematic diagram of rapamycin treatment for behavior tests and spine analysis. Summaries of total dendritic length (C) and Sholl analysis of dendritic complexity (D) of GFP+ neurons show a rescue of normal dendritic arbor length and complexity with Iso/R. Values represent mean ± SEM (*p < 0.05, **p < 0.01; ANOVA for C; *p < 0.0001 ANOVA for D). Numbers in each bar represent number of cells analyzed per group, minimum of 5 animals per group). Summaries of object-place recognition test (E) and Y-maze test (F) for Iso/V and Iso/R show a recovery to near control performance with Iso/R. (Control *n* = 10, Iso/V *n* = 11, Iso/R *n* = 11; *: *p* < 0.05; **: *p* < 0.01, Student *t* test). (G) Representative confocal images of dendritic spines at P60. Scale bar: 2 μm. Shown on right are summary plots of total and mature dendritic spine density. Numbers associated with bar graph indicate the number of dendritic segments examined, a total of 2,586 spines in the control group, 1,831 spines in the isoflurane plus vehicle group, and 2,999 spines in the isoflurane plus rapamycin group were analyzed (****p < 0.0001; ns: non-significant; ANOVA, numbers in each bar represent n of dendritic segments analyzed per group, minimum of 5 animals per group). Underlying data in S1 Data under Fig 3A-G.

A common feature shared by several neurodevelopmental disorders with phenotypes reminiscent of what we have observed in neurons exposed to anesthesia during development is an alteration in signaling in the mTOR pathway [44]. To determine whether activity in the mTOR system is altered by an early exposure to anesthetics we conducted quantitative fluorescence immunohistochemistry using an antibody against phospho-S6 (pS6), a reliable reporter of activity in this pathway [37]. We exposed mice to isoflurane at 1.5% for 4 hours and measured pS6 immunoreactivity in the DGC layer. We found an increase of greater than 2-fold in pS6 intensity at P30 (*p* < 0.0005; Fig 3A, S3 Fig), which was still evident at P60 (S4 Fig). This demonstrates a substantial and lasting upregulation of activity in the mTOR pathway in the dentate gyrus during the period in which we have observed morphological alterations.

We next asked whether increased activity in the mTOR pathway is required for the isoflurane-induced deficits in spatial learning that we observed previously. Mice were exposed to isoflurane 1.5% for 4 hours on P18, given intraperitoneal (IP) injections either of vehicle control or 20 mg/kg rapamycin, a pharmacologic inhibitor of mTOR, every other day between P21 and P29, and then assayed for spatial learning via behavioral testing (Fig 3B). To confirm that our rapamycin treatment effectively suppressed isoflurane-mediated activity in the mTOR pathway, we tested for pS6 immunoreactivity in the dentate gyrus of animals exposed to isoflurane and then treated with rapamycin. We found that rapamycin treatment significantly reduced pS6 immunoreactivity compared to isoflurane and that levels were comparable to untreated controls (Fig 3A).

Subsequently, we tested whether blocking mTOR activation induced by isoflurane could rescue the morphological disruptions and behavioral deficits observed after isoflurane treatment. First, we tested the effects of mTOR inhibition on isoflurane-induced dendrite growth acceleration. We found that rapamycin treatment after isoflurane significantly reduces total dendritic length compared with the control group (*p* < 0.05) and that dendritic length in the isoflurane plus rapamycin group is not significantly different from controls (Fig 3C). Sholl analysis indicates that rapamycin treatment after isoflurane results in arbor complexity that is more similar to what is measured with control conditions than with isoflurane alone (Fig 3D). Rapamycin treatment alone has no effect on spatial learning (S5A–S5D Fig), but rapamycin treatment after isoflurane exposure restores performance to near control levels in both the object-place recognition and Y-maze tests (Fig 3E and 3F and S5C–S5F Fig). Subsequently, we assayed the numbers of dendritic spines in the retrovirus-labeled DGCs exposed to isoflurane with and without rapamycin treatment. We find no significant difference in the total dendritic spine density between the vehicle and rapamycin groups exposed to isoflurane (Fig 3G and S5G and S5H Fig). However, when only the mushroom spines are considered, we find an increase in spine density in the rapamycin group compared to the vehicle treated group (*p* < 0.0001) (Fig 3G). There is no significant difference in mushroom spine density between the control group that did not receive isoflurane and the isoflurane plus rapamycin group (Fig 3G). By contrast stubby spine density appears to be reduced by isoflurane and rapamycin treatment relative to isoflurane alone, and no significant differences are measured in thin spines (S5I and S5J Fig). Thus, our data suggest that rapamycin, by inhibiting the mTOR pathway, prevents an isoflurane-induced reduction in stable synaptic connections.

Taken together, our findings indicate that isoflurane causes a sustained increase in activity in the mTOR pathway that leads to dendrite growth acceleration and either synapse loss or reduced synapse formation in DGCs. Superficially, our results are at odds with a previous study, showing no activation of mTOR in the hippocampus after sevoflurane anesthesia [45], but in the other study, measurements were taken hours after exposure, whereas in the current study we made measurements 1 to 2 weeks later, with a goal of elucidating longer term effects on neuronal development. The mTOR pathway is an intriguing potential mechanism of injury, as it has been implicated both in normal functions in brain development and it is disarrayed in a wide-range of human neurodevelopmental disease [46]. The mTOR pathway is involved in normal development of dendrites and synapses through its actions, integrating signals from the phosphoinositide 3 kinase-protein kinase B (PI3K-Akt) system, which is influenced by both activity and neurotrophic growth factors, such as brain-derived neurotrophic factor (BDNF), that act via tyrosine kinase receptors [47,48]. Downstream mediators of mTOR that influence synaptogenesis include actions on mitochondrial function, lipid synthesis, and translational control via the mTOR1 complex and RhoGTPase actions on the cytoskeleton via the mTOR2 complex [47,48]. Enhanced activity in the mTOR pathway induced by knockdown of disrupted in schizophrenia 1 (DISC1) in newly generated DGCs in adult animals causes accelerated development of dendrites, similar to what we have seen, but it is accompanied by an increase in spine formation [37,49], which stands in apparent contrast to the spine decrease seen in our model. However, several key differences exist between the models that may explain this discrepancy: (1) our study follows the neurons in question for a much longer period, and thus it is possible that overgrowth leads to spine loss over a sufficient length of time; (2) in the DISC1 study, only the studied cohort of newborn DGCs was affected, whereas in our model isoflurane may exert an effect on the surrounding cells as well as the labeled cells; (3) the influence of the DISC1 knockdown was permanent, whereas in our model isoflurane is given transiently and its effects may therefore be manifested differently over time; and (4) we observe overgrowth at P30, which is no longer apparent at P60, and it is possible that early acceleration of growth followed by slowing may induce synaptic loss as a result of a disruption of the normal timing of dendritic arbor growth relative to dendritic spine growth. Additionally, it should be noted that the effects of changes in mTOR signaling may depend on context and on activity in other systems. For instance, Kumar et al. showed that transient inhibition of mTOR, which alone decreases spine formation, could actually increase formation of mushroom spines in a developmental model when it was accompanied by activation of the PI3K-Akt system or treatment with BDNF [50]. In this model, an increase in mushroom spines is accompanied by a decrease in filopodial protrusions that the authors interpret as a destabilization or regression of synapses. Isoflurane and other anesthetics act on multiple targets in developing neurons, and thus understanding their actions on spine and synapse formation will require a full investigation of how each component of the signaling systems that underlie this process is affected.

Given the complexity of the mTOR pathway, the effects of a lasting change in the activity of this pathway are difficult to predict. A sustained increase in mTOR pathway tone certainly has the potential to powerfully alter neurotransmission in the dentate gyrus, as evidenced by the appearance of epileptiform activity in mice with selective deletions of phosphatase and tensin homolog, an mTOR pathway inhibitor, in DGCs [51]. Thus, we hypothesize that isoflurane-induced changes in mTOR signaling have the potential to disrupt the course of neuronal development in the dentate gyrus and perhaps in other brain areas in such a way as to disrupt cognitive function. Even if our findings do not generalize to other cell types and brain regions, they still have significant implications given that substantial populations of DGCs are generated in rodents [52], nonhuman primates [53], and humans [52] during the hypothesized period of susceptibility to anesthesia-induced cognitive deficits in each of these species and these neurons are critical for learning across species. Furthermore, our findings suggest the possibility that harmful effects of mTOR overactivation could be prevented. Complex neurodevelopmental cognitive disorders like autism, in which the pathophysiology may involve changes in mTOR pathway activity that stem from a combination of genetic and environmental factors occurring at unknown times during development, present great challenges in designing an mTOR targeted therapy [54]. By contrast, anesthetic effects on cognitive function result from a brief toxic insult at a known time, and therefore might be more amenable to treatment. Thus, our discovery of a novel, reversible mechanism of injury in developmental anesthetic neurotoxicity has translational potential that can be explored in future studies.

## Methods

### Ethics

All study protocols involving mice were approved by the Animal Care and Use Committee at the Johns Hopkins University (protocol MO14M315) and conducted in accordance with the NIH guidelines for care and use of animals.

### Animals

C57BL/6 mice were housed in a temperature- and humidity-controlled room with a 12:12 hour light:dark cycle, and provided with ad libitum access to water and food. Both sexes were equally represented in all experiments. No animals were excluded.

### Isoflurane treatment and physiologic monitoring of sentinel animals

P18 mouse littermates were randomly assigned to 2 groups. In Group 1 (isoflurane), mice were exposed to 1.5% isoflurane carried in 100% oxygen for 4 hours. A calibrated flowmeter was used to deliver oxygen at a flow rate of 5 L/min and an agent-specific vaporizer was used to deliver isoflurane. In Group 2 (control), mice were exposed to room air for 4 hours. Animals were returned to their cages together with their littermates upon regaining righting reflex. Mice were continually monitored and recorded for skin temperature, heart rate, and oxygen saturation during the 4-hour isoflurane treatment (PhysioSuite; Kent Scientific, Torrington, CT). Intracardiac puncture was used to collect left ventricular blood samples from selected sentinel animals, and those confirmed to be arterial are reported.

### Production and stereotaxic injection of engineered retroviruses

Engineered self-inactivating murine retroviruses were used to express GFP under Ubiquitin promotor (pSUbGW vector) specifically in proliferating cells and their progeny [55,56]. High titers of engineered retroviruses (1 x 10^9^ unit/ml) were produced by cotransfection of retroviral vectors and VSVG into HEK293gp cells followed by ultracentrifugation of viral supernatant as previously described [24,49,55–57]. After induction with a single ketamine injection (50mg/kg), high titers of GFP-expressing retroviruses were stereotaxically injected into the P15 mice dentate gyrus through a 32-gauge microsyringe (Hamilton Robotics, Reno, NV) at 2 sites of the following coordinates relative to the bregma (mm): AP: −2.2, ML: ±2.2, DV: −2.4. The retrovirus-containing solution was injected at a rate of 0.025 μl/min for a total of 0.5 μl per site. After infusion, the microsyringe was left in place for an additional 5 minutes to ensure full virus diffusion and to minimize backflow. After surgery, mice were monitored for general health every day until full recovery. In order to test for a possible confound related to the use of ketamine anesthesia, pS6 immunoreactivity in the dentate gyrus was quantified at P30 in naïve control animals and compared to pS6 immunoreactivity in animals doses with ketamine as above. No significant difference is seen in pS6 levels between these groups (S6 Fig).

### Immunostaining

After transcardial perfusion fixation with 4% paraformaldehyde/PBS, brains were sliced transversely (50 μm thick) with microtome and processed for immunohistochemistry. Primary antibodies, including goat anti-GFP (Rockland, 1:1000) and chicken anti-GFP (Millipore, 1:1000) were used. Immunofluorescence was performed with a combination of Alexa Fluor 488- or Alexa Fluor 594-labeled anti-goat, anti-chicken, or anti-rabbit secondary antibodies (1:250) and 4ʹ,6ʹ-diaminodino-2-phenylindole (DAPI, 1:5000). For analysis of pS6 levels, primary antibodies against pS6-Ser235/236 (rabbit, 1:1000, Cell Signaling) were used. Effective immunostaining of pS6 required an antigen retrieval protocol as previously described [58]. Briefly, sections were incubated in target retrieval solution (DAKO) in 85°C for 20 minutes followed by washing with PBS for t3 times before the incubation with primary antibody.

### Imaging and analyses

Images were acquired on a confocal system (Zeiss LSM 710 or Leica SPE) and morphological analyses were carried out as previously described [24,49,55,56,58,59]. Images for dendritic and spine morphology were deconvoluted with Auto Quant X (Media Cybernetics, Rockville, MD) using the blind algorithm, which employs an iteratively refined theoretical PSF. No further processing was performed prior to image analysis. For visualization, brightness, and contrast levels were adjusted using Image J (NIH). For analysis of dendritic development, three-dimensional (3D) reconstructions of entire dendritic processes of each GFP+ neuron were obtained from Z-series stacks of confocal images using excitation wavelength of 488 nm at high magnification (x 40 lens with 0.7x optical zoom). The two-dimensional (2D) projection images were traced with NIH Image J plugin, NeuronJ. All GFP^+^ DGCs with largely intact, clearly identifiable dendritic trees were analyzed for total dendritic length. The measurements did not include corrections for inclinations of dendritic process and therefore represented projected lengths. Sholl analysis for dendritic complexity was carried out by counting the number of dendrites that crossed a series of concentric circles at 10 μm intervals from the cell soma using ImageJ (NIH). For complete 3D reconstruction of spines, consecutive stacks of images were acquired using an excitation wavelength of 488 nm at high magnification (x 63 lens with 5x optical zoom) to capture the full depth of dendritic fragments (20–35 μm long, 40~70 dendritic fragments in each condition analyzed) and spines using a confocal microscope (Zeiss, Oberkochen. Germany). Confocal image stacks were deconvoluted using a blind deconvolution method (Autoquant X; Media Cybernetics, Rockville, MD). The structure of dendritic fragments and spines was traced using 3D Imaris software using a “fire” heatmap and a 2D x–y orthoslice plane to aid visualization (Bitplane, Belfast, UK). Dendritic fragments were traced using automatic filament tracer, whereas dendritic spines were traced by means of an autopath method with the semiautomatic filament tracer (diameter; min: 0.1, max: 2.0, contrast: 0.8). For spine classification, a custom MatLab (MathWorks, Natick, MA) script was used based on the algorithm; stubby: length (spine) <1.5 and max width (head)<mean_width (neck) *1.2; mushroom: max width (head) >mean width (neck) *1.2 and max_width (head) >0.3; if the spine was not classified as mushroom or stubby, it was defined as long-thin. Axonal bouton volume from axonal fragments was measured by using 3D Imaris software and using a magic wand menu (Bitplane, Belfast, UK) after deconvolution. For analysis of pS6 levels, the sections were processed in parallel and images were acquired using the identical settings, (Zeiss LSM 710, 20X lens). Fluorescence intensity was measured within the granular cell layer using ImageJ (NIH) and the value was normalized to background signal in the same image. These data were then subsequently normalized to the area of the dentate gyrus granule layer as defined by DAPI staining. All experiments were carried out in a blind fashion to experimental conditions.

### Behavioral tests

Sixty-day-old mice housed in groups (5 mice per cage) were handled for at least 2 minutes per day for 3 days before the start of the behavioral experiments. All behavioral tests were performed during the light phase of the cycle between 8:00am and 6:00pm. Experimenters were blind to the samples when behavioral tests were carried out and quantified. The numbers of mice per condition are indicated in the figure legends.

#### Object-place recognition test

Object-place recognition was performed as previously described [37]. Briefly, the test was assessed in a 27.5 cm × 27.5 cm × 25 cm opaque chamber with a prominent cue on 1 of the walls. Each mouse was habituated to the chamber for 15 minutes daily for 2 days. During the training phrase, each mouse was allowed to explore 2 identical objects (glass bottle, 2.7 cm diameter, 12 cm height, and colored paper inside) for 10 minutes. The mouse was then returned to its home cage for a retention period of 24 hours. The mouse was reintroduced to the training context and presented with 1 object that stayed in the same position as during training while the other object was moved to a new position. Movement and interaction with the objects was recorded with a video camera that was mounted above the chamber and exploratory behavior was measured by a blinded observer. Exploratory behavior was defined as sniffing, licking, or touching the object while facing the object.

#### Y-maze test

In the Y-maze test, mice were released from the start arm (no visual cue) and allowed to habituate to only 1 out of 2 possible choice arms (overt visual cue) for 15 minutes. This was followed at 24 hours later by the recognition phrase in which the animal could choose between the 2 choice arms after being released from the start arm. The timed trials (5 minutes) were video recorded as well as graded by an observer blind to condition for total exploration time in each choice arm.

### Rapamycin treatment

P21 mouse littermates were given IP injections of rapamycin (Sigma-Aldrich, St. Louis, MO) prepared from a stock solution (25 mg/ml in 100% ethanol, stored at -20°C) diluted to a final concentration of 4% (v/v) ethanol in the vehicle. Vehicle consisted of 5% Tween 80 (Sigma-Aldrich, St. Louis, MO) and 10% polyethylene glycol 400 (Sigma-Aldrich, St. Louis, MO) as previously described [58,60,61]. Both rapamycin- and vehicle-treated mice received the same volume for each injection (200 μl). Mice received treatments at 48 hour intervals from P21 to P29.

### Statistics

Results are expressed as mean ± SEM. A one-tailed Student *t* test or ANOVA with Bonferroni test for intergroup comparisons were used for most statistical comparisons between groups as described in the figure legends using Prism Software (Graphpad Software Inc, La Jolla, CA). For Sholl analysis ANOVA was used at each point to test for differences between distributions. All data examined with parametric tests were determined to be normally distributed, and the criteria for statistical significance was set a priori at *p* < 0.05. Sample sizes were predicted based on experience from previous similar work [24]. All relevant data are available from the authors.

## Supporting information

S1 Fig A dense field of isoflurane and control group dendrites is shown for P30 and P60 to illustrate the overgrowth phenonenom (scale bar: 50μm). **(A)**. Neurolucida tracings of P60 neurons suggest that the overgrowth does not persist at P60 (scale bar: 20μm) **(B)**, and quantitative analysis by dendrite length measurement **(C)** Sholl analysis **(D)** do not show significant differences between control and isoflurane groups at P60. Isoflurane exposure does not substantially alter DGC distribution. The bar graph in **E** shows positioning of control and isoflurane-exposed newborn DGCs in the dentate gyrus at P30 and P60. Layers 1, 2, and 3 refer to the inner, middle, and outer layers of granule cells in the dentate gyrus, respectively; layer 4 refers to the molecular layer. Soma size of DGCs is significantly increased at P30 (*: p<0.01 Student’s t-test), but not at P60 as show in **F**. To determine whether isoflurane increases branch number, we counted branch points in each dendritic arbor of the labeled neurons. No significant difference was found at either P30 or P60 (**G**). For all bar graphs, numbers on each barindicate the number of neurons examined from at least four mice from per group. Underlying data in S1 Data under Fig S1C-G.
(TIF)

S2 Fig Absolute values for exploration time during the object-place recognition (**A**) and the Y-maze tests (**B**) are shown at 24 h after training (Object place-recognition: Control n = 12, Iso n = 11, *p < 0.05, Student’s t-test; Y-maze: Control n = 12, Iso n = 11; *p < 0.05, Student’s t-test). Individual data points are shown for the object-place recognition (**C**) and Y-maze tasks (**D**) The number of spines counted as a function of the length of each dendritic fragment on which they were counted is represented graphically for total spines (**E**) and mushroom spines (**F**) for the isoflurane (red) and control (black) groups. Dendritic spine density measurements for stubby and thin spine morphologies in control and isoflurane conditions are shown in (**G**) and (**H**), respectively. No significant differences were seen in either of these morphological groups. A reduction in stubby spine density is seen with isoflurane and a further reduction with isoflurane and rapamycin, and no significant difference is measured in thin spine density between any of the conditions (** p < 0.01 ***p < 0.001 ANOVA).
(TIF)

S3 Fig A tiled reconstruction of a representative confocal image of an entire dentate gyrus at P40 with immunohiostochemistry for pS6 and counterstaining for DAPI for control and isoflurane-exposed is shown (blue: DAPI; red: pS6). Scale bar: 100 μm.
(TIF)

S4 Fig Immunoreactivity for pS6 measured at P60 is significantly increased after isoflurane treatment, and is reduced with rapamycin treatment. (***p < 0.001, ****p < 0.0001, ANOVA, numbers in each bar represent n for images analyzed) Scale bar: 50 μm. Underlying data in S1 Data file under Fig S4.
(TIF)

S5 Fig As an additional control tests of spatial learning were performed on animals treated with rapamycin only, in the absence of isoflurane to test for possible effects or rapamycin independent of anesthesia-induced deficits. No change in performance relative to control is measured with rapamycin in either object place-recognition (**A**) or Y-maze test paradigms (**B**). These results are also presented in the context of the control, isoflurane, and isoflurane plus rapamycin groups (**C**,object place recognition; **D**, Y-maze). Individual data points are shown for the object-place recognition (**E**) and Y-maze tasks (**F**). The number of spines counted as a function of the length of each dendritic fragment on which they were counted is represented graphically for total spines (**G**) and mature spines (**H**) for the isoflurane (red) and control (black) groups. Dendritic spine density measurements for stubby and thin spine morphologies in control and isoflurane conditions are shown in (**I**) and (**J**), respectively. Underlying data in S1 Data under Fig S5A-J.
(TIF)

S6 Fig All animals used in experiments requiring stereotaxic injection of retrovirus, including both controls and isoflurane exposed groups, were anesthetized with small doses of ketamine to facilitate the surgery. To test for a possible confounding effect of ketamine, levels of pS6 labeling in the dentate gyrus were measured in naïve controls and in animals that received ketamine only. No significant difference pS6 immunoreactivity is seen between the two groups (Student’s t-test). Numbers on each bar indicate the number for images analyzed from at least five mice from per group. Scale bar: 25 μm. Underlying data in S1 Data under Fig S6.
(TIF)

S1 Table Data describing the physiologic response to anesthesia from is presented from a cohort of sentinel animals. As in experimental protocols, mouse pups on postnatal day 18 (P18) were induced with Isoflurane 3% in oxygen until loss of righting reflex, and anesthesia was maintained at 1.5% in oxygen for 4h while the animals were spontaneously ventilating. Heart rate, oxyhemoglobin saturation, and skin surface temperature were measured with the Kent Scientific PhysioSuite hourly, and values obtained throughout a given hour were averaged **(T1A)**. Data are presented in **T1A** as the mean ± SEM (n = 4 readings taken in 6 sentinel animals). At the end of the protocol animals were sacrificed, and blood samples were obtained by attempted cannulation of the left ventricle. Due to technical limitations we were not able to obtain an arterial sample for all animals. The value for partial pressure of oxygen for arterial samples is shown as is the blood glucose concentration for all samples **(T1B)**. Underlying data in S1 Data under Fig 3A–3G. Underlying data in S1 Data under Fig S1T.
(TIF)

S1 Data Source data. Cited in figure legends in manuscript.
(XLSX)

## Abbreviations

BDNF: brain-derived neurotrophic factor
DCG: dentate gyrus granule cell
DIV: day in vitro
DISC1: disrupted in schizophrenia 1
GFP: green florescent protein
IP: intraperitoneal
mTOR: mechanistic target of rapamycin
P: postnatal day
PI3K-Akt: phosphoinositide 3 kinase-protein kinase B
pS6: phosphor-S6
