# Correction: Early postnatal exposure to isoflurane causes cognitive deficits and disrupts development of newborn hippocampal neurons via activation of the mTOR pathway

**DOI:** 10.1371/journal.pbio.1002625

**Published:** 2018-03-29

**Authors:** The PLOS Biology Staff

The Academic Editor’s name and affiliation information was omitted from the original published article. The publisher apologizes for the error. The Academic Editor’s name and affiliation is as follows: Lisa Monteggia, UT Southwestern Medical Center, United States of America.
