# Supplementary figures and images for: Early postnatal exposure to isoflurane causes cognitive deficits and disrupts development of newborn hippocampal neurons via activation of the mTOR pathway
Source: PLoS Biol. 2017 Jul 6;15(7):e2001246. doi: 10.1371/journal.pbio.2001246 (PMC5500005; doi:10.1371/journal.pbio.2001246)

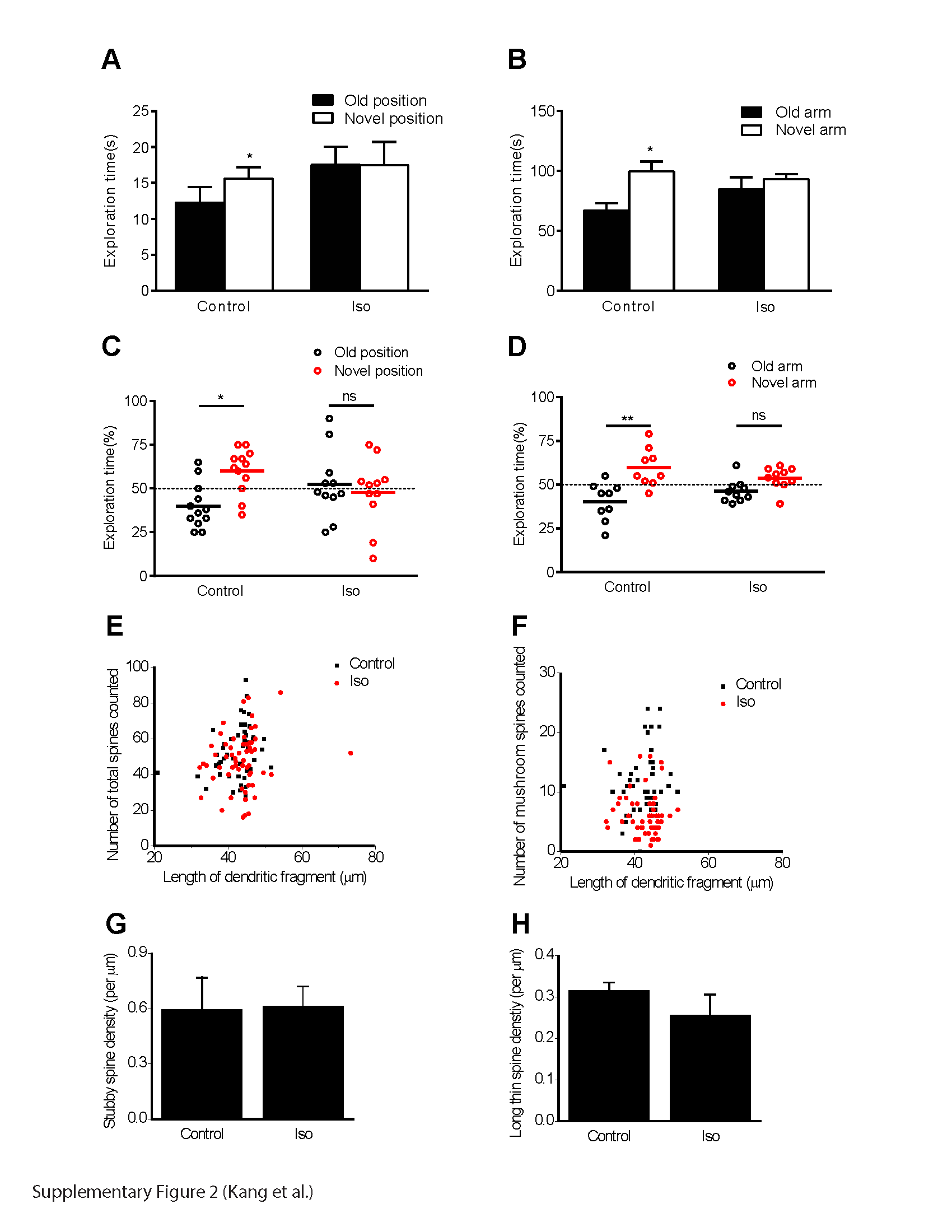

Supplement: S2 Fig — Absolute values for exploration time during the object-place recognition (A) and the Y-maze tests (B) are shown at 24 h after training (Object place-recognition: Control n = 12, Iso n = 11, *p < 0.05, Student’s t-test; Y-maze: Control n = 12, Iso n = 11; *p < 0.05, Student’s t-test). Individual data points are shown for the object-place recognition (C) and Y-maze tasks (D) The number of spines counted as a function of the length of each dendritic fragment on which they were counted is represented graphically for total spines (E) and mushroom spines (F) for the isoflurane (red) and control (black) groups. Dendritic spine density measurements for stubby and thin spine morphologies in control and isoflurane conditions are shown in (G) and (H), respectively. No significant differences were seen in either of these morphological groups. A reduction in stubby spine density is seen with isoflurane and a further reduction with isoflurane and rapamycin, and no significant difference is measured in thin spine density between any of the conditions (** p < 0.01 ***p < 0.001 ANOVA). (TIF) [file pbio.2001246.s002.tif]

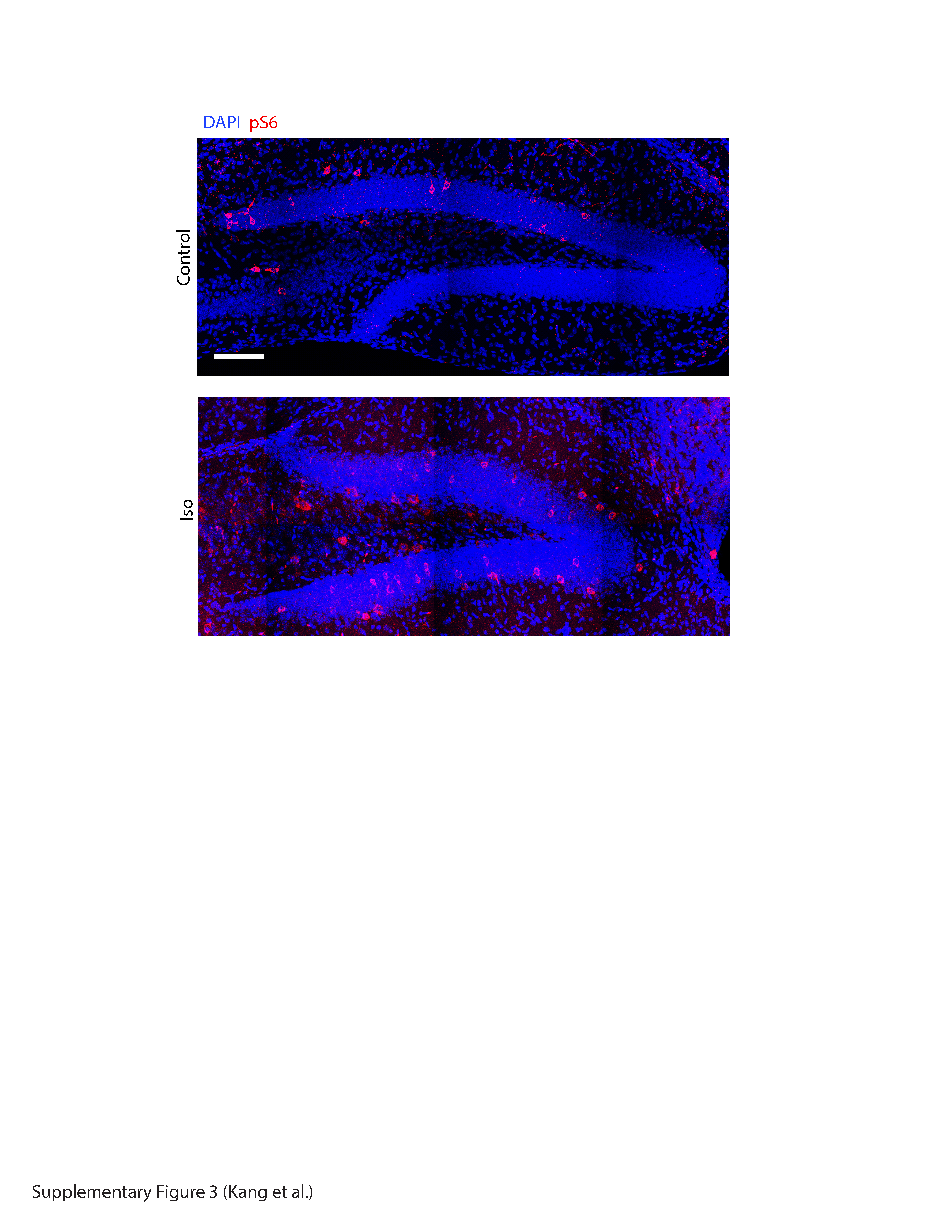

Supplement: S3 Fig — Scale bar: 100 μm. (TIF) [file pbio.2001246.s003.tif]

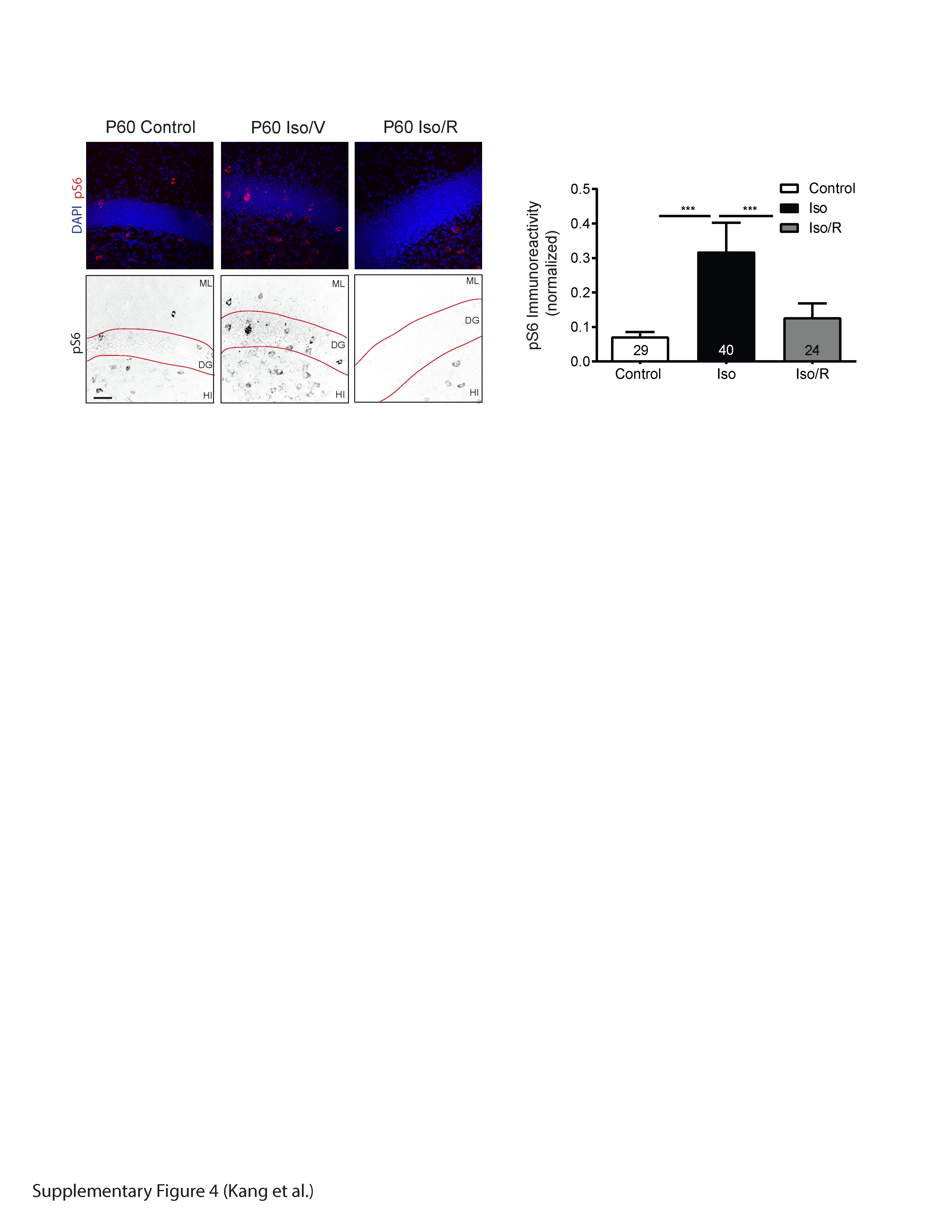

Supplement: S4 Fig — (***p < 0.001, ****p < 0.0001, ANOVA, numbers in each bar represent n for images analyzed) Scale bar: 50 μm. Underlying data in S1 Data file under Fig S4. (TIF) [file pbio.2001246.s004.tif]

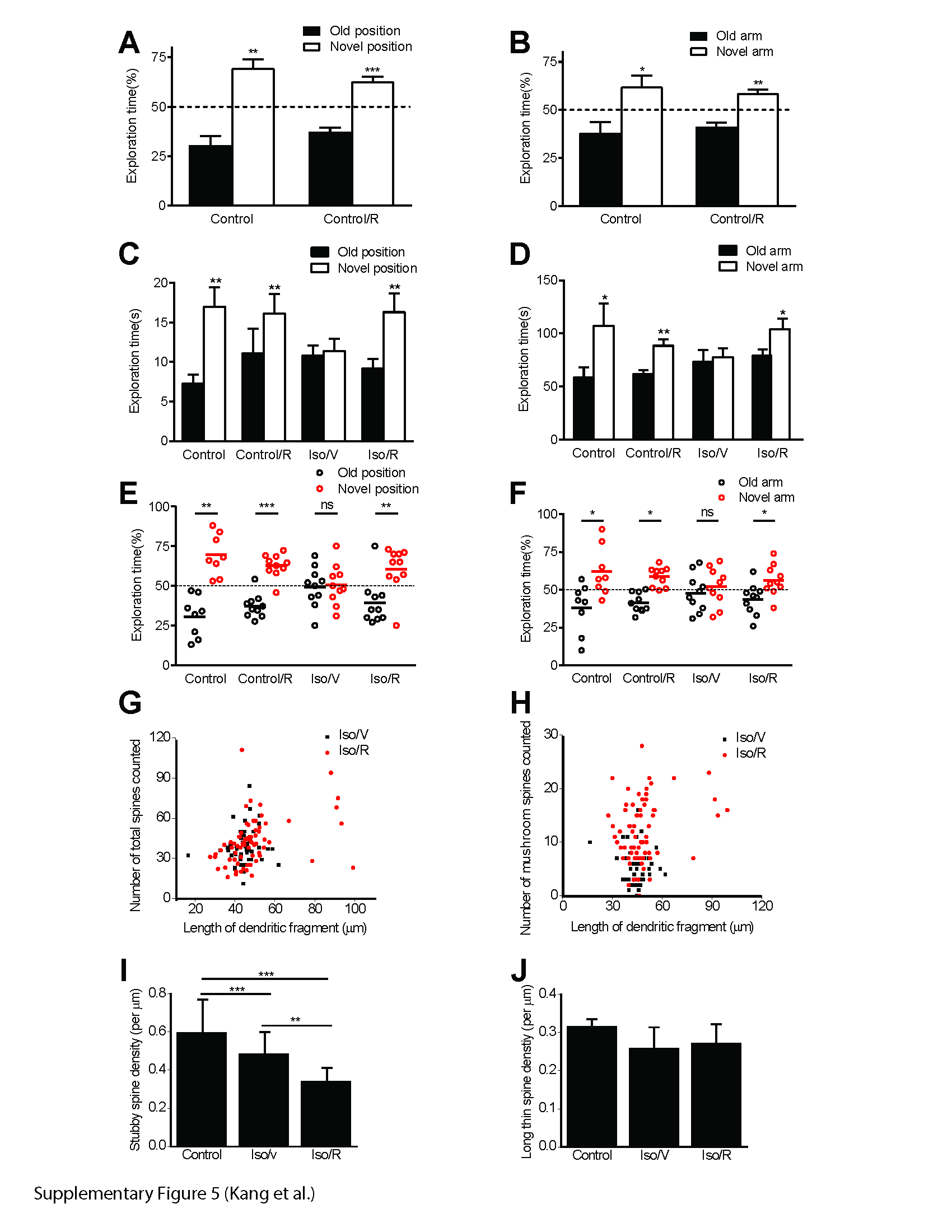

Supplement: S5 Fig — No change in performance relative to control is measured with rapamycin in either object place-recognition (A) or Y-maze test paradigms (B). These results are also presented in the context of the control, isoflurane, and isoflurane plus rapamycin groups (C,object place recognition; D, Y-maze). Individual data points are shown for the object-place recognition (E) and Y-maze tasks (F). The number of spines counted as a function of the length of each dendritic fragment on which they were counted is represented graphically for total spines (G) and mature spines (H) for the isoflurane (red) and control (black) groups. Dendritic spine density measurements for stubby and thin spine morphologies in control and isoflurane conditions are shown in (I) and (J), respectively. Underlying data in S1 Data under Fig S5A-J. (TIF) [file pbio.2001246.s005.tif]

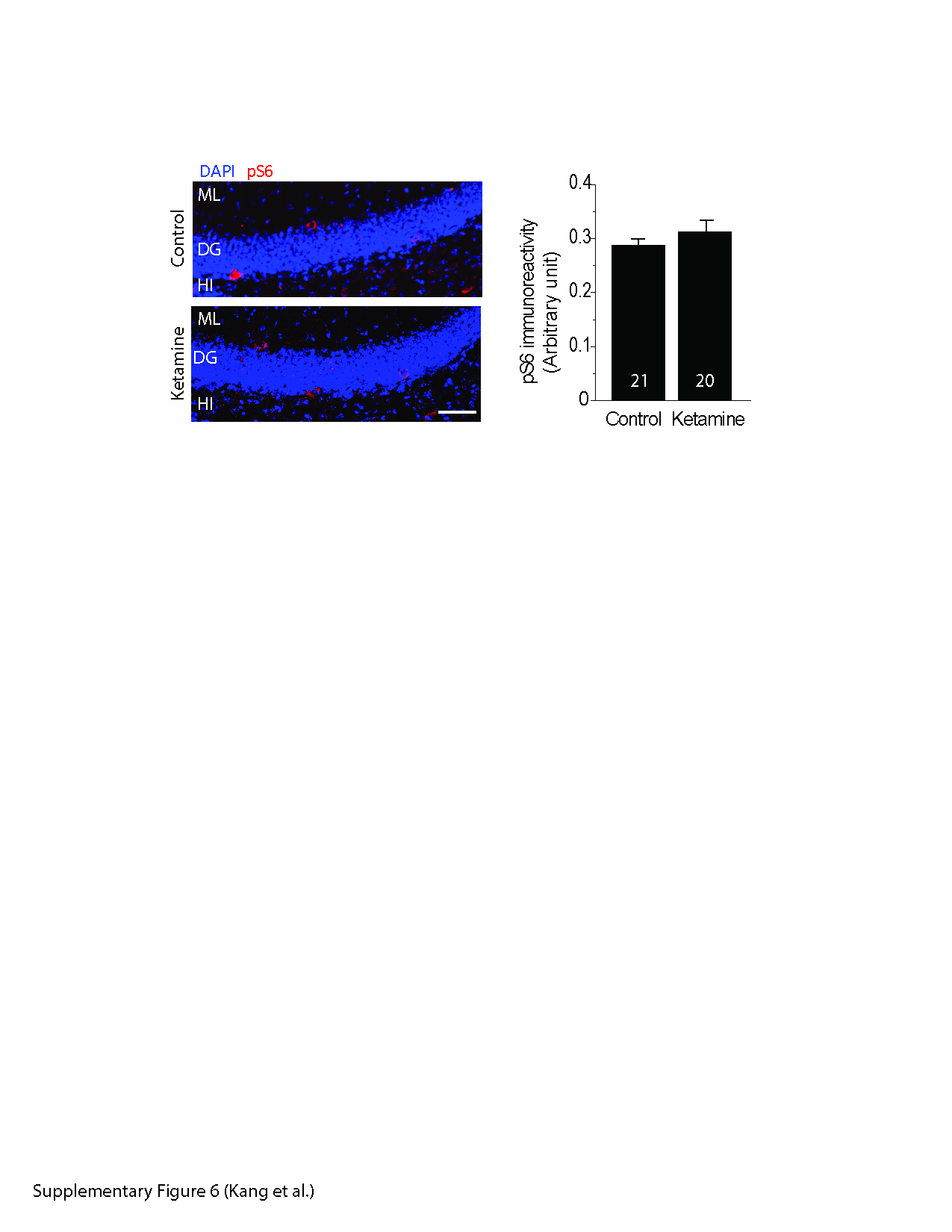

Supplement: S6 Fig — To test for a possible confounding effect of ketamine, levels of pS6 labeling in the dentate gyrus were measured in naïve controls and in animals that received ketamine only. No significant difference pS6 immunoreactivity is seen between the two groups (Student’s t-test). Numbers on each bar indicate the number for images analyzed from at least five mice from per group. Scale bar: 25 μm. Underlying data in S1 Data under Fig S6. (TIF) [file pbio.2001246.s006.tif]

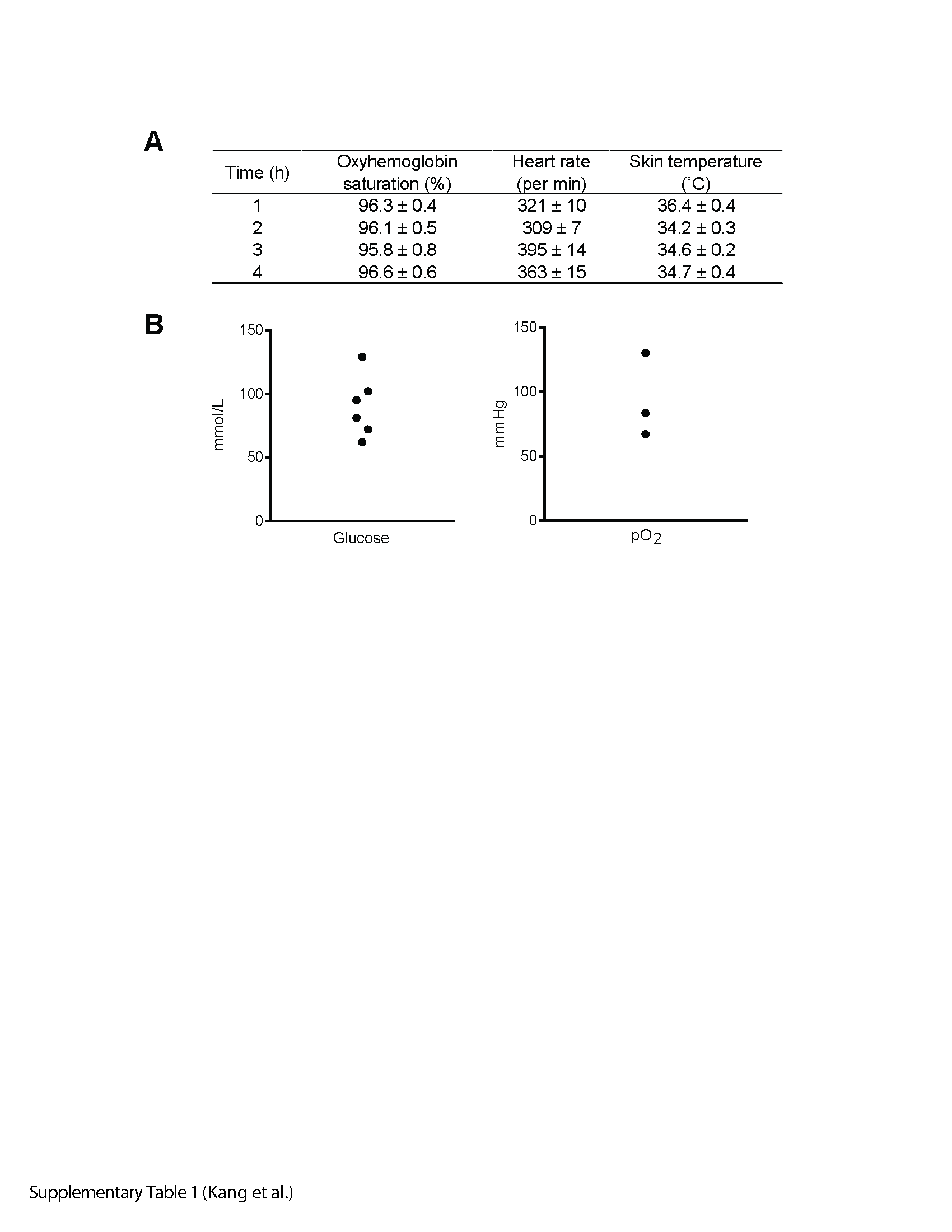

Supplement: S1 Table — As in experimental protocols, mouse pups on postnatal day 18 (P18) were induced with Isoflurane 3% in oxygen until loss of righting reflex, and anesthesia was maintained at 1.5% in oxygen for 4h while the animals were spontaneously ventilating. Heart rate, oxyhemoglobin saturation, and skin surface temperature were measured with the Kent Scientific PhysioSuite hourly, and values obtained throughout a given hour were averaged (T1A). Data are presented in T1A as the mean ± SEM (n = 4 readings taken in 6 sentinel animals). At the end of the protocol animals were sacrificed, and blood samples were obtained by attempted cannulation of the left ventricle. Due to technical limitations we were not able to obtain an arterial sample for all animals. The value for partial pressure of oxygen for arterial samples is shown as is the blood glucose concentration for all samples (T1B). Underlying data in S1 Data under Fig 3A–3G. Underlying data in S1 Data under Fig S1T. (TIF) [file pbio.2001246.s007.tif]
